# Comparison of Standing Side Bending Using Biplanar Stereography and Fulcrum Bending for Flexibility Assessment of Adolescent Idiopathic Scoliosis

**DOI:** 10.3390/jcm13216370

**Published:** 2024-10-24

**Authors:** Andreas Frodl, Tanja Wendling, Lukas Klein, Ferdinand C. Wagner, Nils Mühlenfeld, Benjamin Erdle, Moritz Mayr, Thomas Niemeyer, Peter Obid

**Affiliations:** 1 Department of Orthopedic Surgery and Traumatology, Freiburg University Hospital, Albert Ludwigs University Freiburg, Hugstetter Straße 55, 79106 Freiburg, Germanypeter.obid@uniklinik-freiburg.de (P.O.); 2 Asklepios Klinik Wiesbaden, Spine Center, Geisenheimer Str. 10, 65197 Wiesbaden, Germany

**Keywords:** idiopathic adolescent scoliosis (AIS), fulcrum bending, stereography

## Abstract

**Background**: The surgical treatment of adolescent idiopathic scoliosis (AIS) is influenced by factors such as skeletal maturity, curve magnitude, progression, and spinal flexibility. The assessment of spinal flexibility is crucial for surgical planning; supine bending radiographs are commonly used but there is no consensus on the optimal technique. Fulcrum bending radiographs (FBRs) have shown better prediction of post-surgery correction compared to supine bending radiographs. New radiological techniques allow a significant reduction in radiation exposure. This study aims to compare spinal flexibility assessment and radiation exposure between FBRs and standing side-bending radiographs (SSRs) using biplanar stereoradiography. **Materials and Methods**: Twenty-five consecutive AIS patients scheduled for surgery were included in this prospective cohort study. Exclusion criteria were non-idiopathic etiology, age younger than 12 years, and older than 18 years. Pre-surgery FBRs and SSRs were performed, and the Cobb angles were measured. Flexibility and correction rates were calculated. Dose–area products (DAPs) were recorded. Statistical analysis was conducted using the Wilcoxon signed-rank test and Spearman correlation. **Results**: The mean pre-surgery Cobb angle of the thoracic curve was 66.3°. The FBR was superior to SSR for assessing flexibility for thoracic curves and provided a better prediction for post-surgery correction. There was no significant difference in lumbar curves between FBR and SSR. The mean DAP for SSRs was 0.81 Gy*cm^2^ compared to 3.42 Gy*cm^2^ for FBR, indicating a lower radiation exposure using SSRs. **Conclusions**: FBRs are superior for flexibility assessment of thoracic curves in AIS and offers a better prediction of post-surgery correction compared to SSRs. However, FBR is associated with a higher radiation exposure.

## 1. Introduction

The surgical management of adolescent idiopathic scoliosis (AIS) is a decision-making process influenced by several critical factors, including the patient’s skeletal maturity, the magnitude and progression of the scoliotic curve, and the flexibility of the curve. Surgical intervention typically involves spinal fusion, and the choice of the levels to be included in the fusion area is determined by these parameters. While there are no definitive criteria for selecting fusion levels, the widely accepted Lenke classification provides a well-established framework that aids surgeons in determining the upper and lower instrumented vertebrae for posterior spinal fusion (PSF) procedures [1]. The classification aims to guide surgeons regarding their selection of upper and lower instrumented vertebrae that should be included in a posterior spinal fusion (PSF). Flexibility assessment is critical to surgical planning, as it informs the surgeon about the degree to which the scoliotic curve may be corrected. It also differentiates structural and non-structural curves. These assessments are typically conducted using a variety of radiographic techniques, such as Fulcrum bending radiographs (FBRs), standing and supine bending radiographs, and traction radiographs [2]. Each of these methods offers insight into the flexibility of the spine, which helps to predict how the curves may respond to surgical correction. Different flexibility radiographs are commonly used in clinical practice, although there is no universal consensus on which method provides the most accurate flexibility measurement [3,4,5]. Supine bending radiographs are frequently employed due to their widespread availability and ease of use, yet there is ongoing debate regarding their efficacy compared to other methods [6]. He et al. literature review reported that fulcrum bending radiographs (FBRs) offered a more accurate assessment of spinal flexibility for thoracic curves and provided a superior prediction of post-surgical correction compared to supine bending radiographs [7]. However, one major drawback of traditional radiographic imaging in AIS patients is the significant radiation exposure, especially when compared to more advanced techniques such as biplanar stereoradiography. This method, which includes both anteroposterior (AP) and lateral projections, has been shown to reduce radiation exposure while providing high-resolution, three-dimensional images of the spine [5,8].

Despite these advancements, there remains a lack of comparative data on the use of different bending techniques, specifically between fulcrum bending radiographs and standing side-bending radiographs (SSRs), when applied in conjunction with biplanar stereoradiography. Therefore, the primary objective of this study was to compare spinal flexibility assessments obtained using FBRs and SSRs, the reliability of correction prediction, and to evaluate the radiation exposure associated with each technique in patients with AIS undergoing surgical correction.

## 2. Materials and Methods

### 2.1. Patients

This prospective cohort study was initiated according to the STROBE guidelines following approval from the local ethics committee (Landesärztekammer Hessen, approval number FF 117/2018). Twenty-five consecutive patients scheduled for surgical correction of AIS were included in the study. The inclusion criteria were limited to individuals aged between 12 and 18 years with a diagnosis of relevant idiopathic scoliosis with an indication for surgical correction (Cobb angle > 40°), while patients with non-idiopathic etiologies or those outside the age range were excluded. Prior to the surgery, all patients received an MRI scan of the whole spine to exclude intraspinal pathologies and secondary scoliosis. The inclusion of the patients was carried out by their legal guardians as informed consent due to the patients’ minority.

### 2.2. Methods

Prior to surgery, full-body standing biplanar stereoradiographs were obtained for all patients using the EOS imaging system (EOS Imaging, Paris, France). For comparison of flexibility assessment, all patients received FBRs of the thoracic and thoracolumbar curves according to Cheung et al. [9] and SSRs using biplanar stereoradiography. Since the anatomical positioning of the fulcrum presents limitations in accurately assessing proximal thoracic curves, this method was primarily utilized for thoracic and thoracolumbar regions.

**Figure 1:**
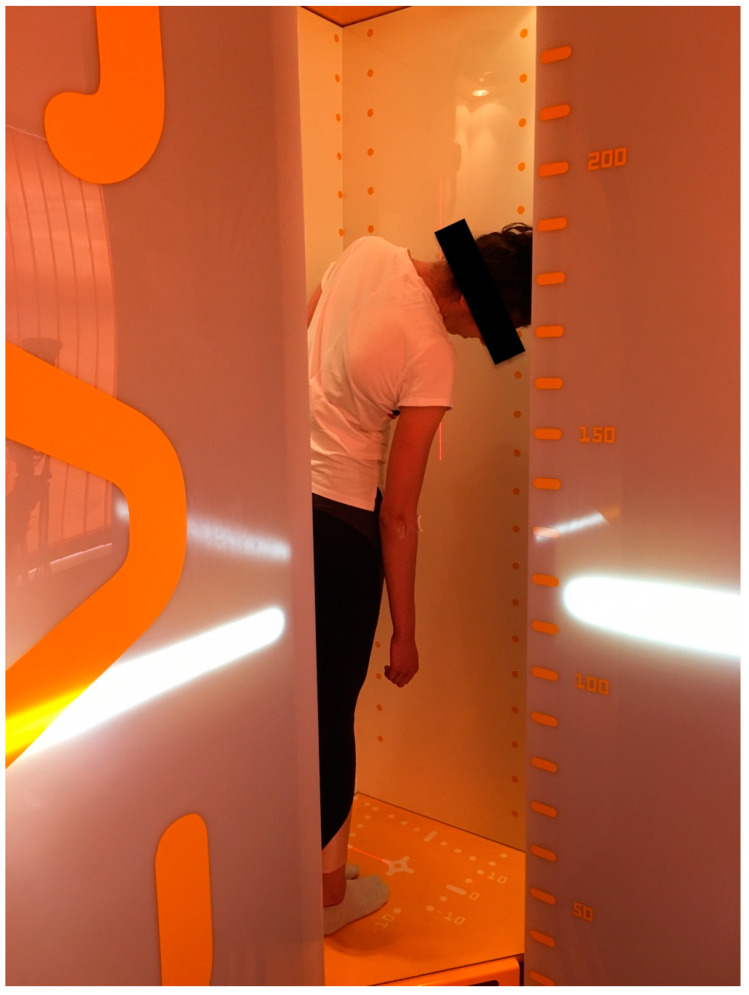
A patient placed in the EOS booth for lateral side bending films.

The second technique involved standing side-bending radiographs (SSRs) performed within the EOS imaging system. During recording of the SSRs, patients stood with their arms relaxed by their sides and were positioned eccentrically within the imaging booth to maximize the range of motion during lateral bending. This positioning enabled the acquisition of only AP radiographs (Figure 1).

### 2.3. Measurements

The radiographic images obtained from pre-surgery FBRs and SSRs were analyzed, and the Cobb angles of the thoracic and thoracolumbar curves were measured in a standardized way [10]. The Cobb angles were determined by the authors before and 6 months after surgical treatment. The imaging material was not blinded; however, care was taken to ensure that the postoperative measurement of the Cobb angles was not performed by the surgeon who had previously operated.

The flexibility of the spine was calculated for both FBRs and SSRs using the following formula:

$$\frac{\left(\mathrm{Pre-surgery}\;\mathrm{Cobb}\;\mathrm{Angle}-\mathrm{Bending}\;\mathrm{Cobb}\;\mathrm{Angle}\right)}{\mathrm{Pre-surgery}\;\mathrm{Cobb}\;\mathrm{Angle}}\times 100\%.$$

The correction rate was calculated using the following formula:


$$\frac{\left(\mathrm{Pre-surgery}\;\mathrm{Cobb}\;\mathrm{Angle}-\mathrm{Post-surgery}\;\mathrm{Cobb}\;\mathrm{Angle}\right)}{\mathrm{Pre-surgery}\;\mathrm{Cobb}\;\mathrm{Angle}}\times 100\%.$$

With the correction rate, a flexibility index was calculated to compare the ability of FBRs and SSRs to predict the post-surgery correction:

$$\frac{\mathrm{Correction}\;\mathrm{Rate}}{\mathrm{Bending}\;\mathrm{Flexibility}}\times 100\%.$$

To evaluate radiation exposure, the dose–area product (DAP), expressed in Gy*cm^2^, was collected from the hospital’s picture archiving and communication system (PACS). The DAP values were recorded and compared for the different bending techniques.

### 2.4. Statistical Analysis

Statistical analysis was performed using the Wilcoxon signed-rank test, appropriate for non-parametric data, to identify significant differences between FBRs and SSRs. The normality of the data was assessed using the Shapiro–Wilk test, and statistical significance was defined as a *p*-value less than 0.05. All analyses were conducted using IBM SPSS Statistics, version 29.0.0.0 (IBM Corp., Armonk, NY, USA). Due to the small sample size, post-hoc power analyses have been performed using G-power (Version 3.1.9.6).

The outcomes of these analyses were interpreted with an exploratory approach, providing insights into the relative merits of the two flexibility assessment methods and their impact on radiation exposure.

## 3. Results

A total of 25 patients (female *n* = 22, male *n* = 3) with AIS were included in the present study. The mean age at the time of surgery was 14.2 ± 1.47 years (range: 12–18 years). The Cobb angles were measured prior to surgery using FBRs and SSRs. A detailed overview of demographic data and Cobb angles are shown in Table 1 and Table 2.

The mean pre-surgery Cobb angle of the main thoracic curve was 66.3° (±19.5). Using FBRs and SSRs, the flexibility of the main thoracic curves resulted in 31.6° (±13.7) and 45.5° (±18.1), respectively. FBRs were compared with SSRs regarding the main thoracic (MT) and thoracolumbar/lumbar curves (TL/L). As depicted in Table 2, the flexibility of the main thoracic curve is superiorly reflected using FBRs. For MT curves, the FBR shows significantly better flexibility and prediction of surgical correction (*p* = 0.001). The difference between FBRs and SSRs was not significant for TL/L curves (*p* = 0.032).

**Figure 2:**
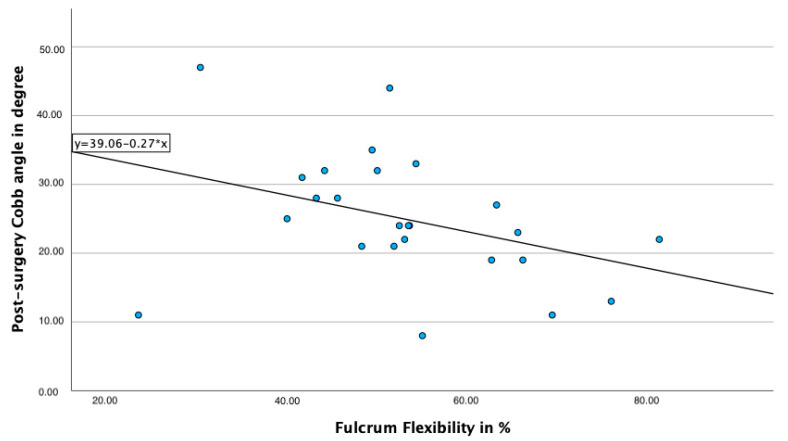
Correlation of Fulcrum flexibility and post-surgery Cobb angle.

Using Spearman correlation, a significant, negative correlation between the post-surgery Cobb angle and Fulcrum bending flexibility is observed (r= −0.48; *p* = 0.014), even though the correlation of these parameters is rather weak (Figure 2). It can be observed that lower fulcrum flexibility correlates with a higher post-surgery Cobb angle.

Correction indexes were calculated for Fulcrum bending and EOS imaging. Results are visualized in Table 3. In a subanalysis, fulcrum flexibility was proven to be a better predictor for post-surgery correction compared to SSR flexibility (Table 4).

In addition to the correction rate and flexibility index, the dose–area product of the resulting ionizing radiation was determined depending on the type of imaging. The mean dose–area product (DAP) for SSRs using EOS imaging was calculated with 0.81 Gy*cm^2^ (±0.38), whereas the mean DAP for FBRs was 3.42 Gy*cm^2^ (±2.09).

Due to the small study population of only 25 patients that could be recruited during the study period, we conducted a post-hoc power analysis to ensure that the findings and results of this study were interpreted correctly. In consideration of a conservative approach to assess effect sizes, the lowest z-value was used to determine the effect size. This value was documented as z = −1.743, resulting in a Cohen’s d of 0.744. Based on this data, we performed the post-hoc power analysis using G-Power. This yielded an underlying statistical power of 0.956.

## 4. Discussion

The main finding of the present study is a significantly better flexibility assessment and prediction of post-surgery correction for main thoracic curves using FBRs compared to SSRs. The flexibility of proximal thoracic curves might not be sufficiently addressed using SSRs since the head of patient might leave the image area of the EOS device when leaning sideways. There was no difference for assessing thoracolumbar curves using FBRs or SSRs. Concerning the radiation exposure, FBRs were shown to result in significantly higher doses compared to SSRs.

Pre-surgery flexibility analysis is an essential step in surgical planning for choosing fusion levels and implant placement. Adequate selection of fusion levels guarantees a stable long-term result [6,11]. Different methods for radiological evaluation exist, but cause a relevant exposure to ionizing radiation, which is a major concern especially in young patients [8,12,13,14,15]. In a retrospective cohort study, Prescutti et al. reported a 1% to 2% increased lifetime risk of developing breast and thyroid cancer as a result of ionizing radiation in adolescent idiopathic scoliosis patients treated before the 1990s [16]. As part of scoliosis therapy, full-spine radiographs in conventional X-rays are an important diagnostic tool regarding the course and treatment planning. However, these images are associated with high radiation exposure in the case of standard X-rays [13,17,18]. More recently developed techniques such as the EOS System are considered radiation-sparing [8]. In 2015, Luo et al. demonstrated a significant reduction in ionizing radiation when using EOS in the examination of 42 pediatric scoliosis patients [17]. However, the long-term health benefits from reduced radiation exposure with EOS still need to be assessed [19,20].

In the application of bending radiographs to determine spinal flexibility, these images are commonly taken either as fulcrum bending radiographs (FBRs) or standing side bending radiographs (SSRs) [5]. Masuda et al. analyzed the predictive value of FBRs and SSRs for post-surgery correction of the major curve. In a consecutive analysis of 25 patients with AIS, the FBR was found to be more predictive for the major scoliotic curve correction [21]. These results are confirmed by our study as well.

In a prospective comparison of four different techniques for pre-surgery flexibility estimation, Klepps et al. demonstrated a better prediction using FBRs [22]. The study by Klepps et al. comprehensively investigated various radiographic techniques for preoperative assessment of curve flexibility in scoliosis, including lying supine, supine side-bending radiographs, fulcrum bending radiographs (FBRs), and push-prone radiographs. The authors found that for the main thoracic curves (MT curves), the FBR method showed a significantly superior correction estimation in preoperative flexibility measurements compared to the other techniques. However, for the thoracolumbar/lumbar curves (TL/L curves), the study did not show significant differences between side-bending radiographs and FBRs in evaluating curve flexibility.

Interestingly, when comparing the preoperatively assessed flexibility with the postoperatively determined Cobb angles, the study found that none of the imaging methods could reliably predict the actual correction in either MT or TL/L curves [21].

Hay et al. used FBRs in a series of 90 patients undergoing anterior scoliosis correction and concluded a high predictive value for FBRs in estimating anterior scoliosis correction [23].

The meaningfulness of our study is clearly hampered by the small number of patients.

In respect of the limited space in the EOS booth, the effect of the patient’s size and weight has to be evaluated. Also, especially in SSRs, a certain level of patient compliance is required to hold on to the bending position, until the image is taken. Thus, “Position incompliance” could be a potential confounder, biasing results.

### Limitations

One limitation of this study is the small sample size, which results in findings of limited generalizability. Additionally, the inclusion of SSRs requires specific patient compliance, as the side-bending position must be consistently maintained for a minimum of 5 s.

## 5. Conclusions

FBRs are superior for flexibility assessment of thoracic curves in AIS and offers a better prediction of post-surgery correction compared to SSRs. However, FBRs are associated with a higher radiation exposure.

## Figures and Tables

**Table 1:** Demographic Data of included patients (standard deviation).

	Total
Number of patients	25
Age in years	14.2 (±1.5) (range: 12–18)
Weight in kg	56.2 (±13.4) (range: 41–86)
Height in cm	167.8 (±8.4) (range: 149–185)

**Table 2:** Overview of the Cobb angles and comparison of bending radiographs.

Cobb Angles in Degree	Pre-Surgery Mean ± SD	Post-Surgery Mean ± SD	Fulcrum Bending Mean ± SD	EOS (SSR) Mean ± SD	*p*
Proximal thoracic (PT)	33.0 ± 14.5°	17.6 ± 8.3°	n/a	23.0 ± 9.5°	n/a
Main thoracic (MT)	66.3 ± 19.5°	25.0 ± 9.4°	31.6 ± 13.7°	45.5 ± 18.1°	0.001
Thoracolumbar/lumbar (TL/L)	46.6 ± 9.3°	17.0 ± 7.0°	14.5 ± 6.7°	12.5 ± 8.2°	0.032

**Table 3:** Overview of correction rates, flexibility rates and correction Index.

	Proximal Thoracic (PT)	Main Thoracic (MT)	Thoracolumbar/Lumbar (TL/L)
Post-surgery Correction rate in %	43.6 ± 16.8	61.3 ± 14.0	63.3 ± 13.4
Fulcrum Bending Radiograph			
Fulcrum Flexibility (%)	n/a	53.0 ± 13.0	68.5 ± 14.7
Fulcrum Bending Correction Index (%)	n/a	122.6 ± 45.2	95.0 ± 20.9
EOS Imaging (SSR)			
SSR Flexibility (%)	27.0 ± 9.8	33.3 ± 16.3	73.0 ± 16.3
SSR Correction Index (%)	134.6 ± 74.6	264.6 ± 199.1	68.3 ± 27.9

**Table 4:** Comparison between Fulcrum flexibility and SSR flexibility.

	Correction Rate	Fulcrum Flexibility	*p*	SSR-Flexibility	*p*
Main thoracic (MT)	61.3 ± 14.0	53.0 ± 13.0	0.001	33.3 ± 16.3	0.001
Thoracolumbar/lumbar (TL/L)	63.3 ± 13.4	68.5 ± 14.7	0.001	73.0 ± 16.3	0.581

## Data Availability

All data are within the manuscript.
